# Management of pulmonary vasodilator therapy in patients with pulmonary arterial hypertension during critical illness

**DOI:** 10.1186/s13054-014-0523-z

**Published:** 2014-10-07

**Authors:** Katie M Muzevich, Hadi Chohan, Daniel C Grinnan

**Affiliations:** Virginia Commonwealth University Health System, 401 N. 12th Street, P.O. Box 980042, Richmond, VA 23298 USA; Virginia Commonwealth University Health System, 1200 East Broad Street, P.O. Box 980050, Richmond, VA 23298 USA

## Abstract

Pulmonary arterial hypertension (PAH) is commonly treated with pulmonary arteriolar vasodilator therapy. When a patient on PAH medication is admitted to intensive care, determining how to manage their medication during the critical illness is often complicated. There may be considerations related to the inability to take medication by mouth, related to acute renal failure or acute liver injury, related to altered mental status or delirium, or related to hypotension and bacteremia. Decisions of how to manage these medications can have a major impact on the patient’s clinical course. Presently, provider experience is the major tool in navigating the decisions regarding these medications. In this review, we offer our recommendations of how to manage PAH patients with critical illness who are on PAH medications. These recommendations include how to deliver medications via feeding tubes, how to dose medications in the setting of acute renal failure or acute liver failure, and how to manage medications during hypotension or when a tunneled catheter needs to be removed.

## Introduction

Pulmonary arterial hypertension (PAH) is a progressive disorder of the pulmonary circulation, which leads to right ventricular failure and death. In the past two decades, advances have led to US Food and Drug Administration approval of several PAH therapies for the treatment of PAH, and their use is now widespread. Common side effects and use of these medications in the outpatient setting have been extensively discussed. However, we are unaware of a review of PAH therapies focusing on management when patients are admitted to an ICU. Many patients with PAH die in an ICU setting [1,2], and the reported prevalence of PAH may have increased from the 1980s, when a National Institutes of Health registry enrolled less than 200 patients [3], to the present, with more than 3,500 patients enrolled in the US-based Registry to Evaluate Early and Long-term PAH Disease Management (REVEAL) [4]. A discussion of how to manage existing PAH therapies in patients admitted to the ICU is therefore overdue. This management includes the use of phosphodiesterase inhibitors, endothelin receptor antagonists and prostacyclin analogs. Our discussion will not include fluid management of PAH patients admitted to the ICU, because this topic has previously been discussed in other publications [5,6].

At our institution, we routinely care for patients on treatment for PAH during inpatient admissions. Often this includes transfer from another hospital. Over the past decade, we average over 30 PAH patients per year admitted with varying conditions. We offer our single-center experience with PAH therapies in the ICU setting, as well as our recommended approach to their use during ICU admission.

## Medication administration during critical illness

Administration of medications may be compromised when patients require mechanical ventilation and enteral nutrition, but it is possible via an enteral feeding tube if the drug formation is suitable for enteral administration. Generally, liquid formulations are preferred over tablet or capsule dosage forms [7]. In the absence of a liquid dosage form, many solid dosage forms (tablets or capsules) can be crushed or opened and mixed with water for enteral administration. Dosage forms that should not be altered for enteral administration include those of extended or delayed release, and drugs with chemotherapeutic, teratogenic or carcinogenic properties. Phosphodiesterase type 5 inhibitors (sildenafil and tadalafil), endothelin receptor antagonists (bosentan, ambrisentan and macitentan) and the novel soluble guanylate cyclase stimulator (riociguat) are oral medications for the treatment of PAH. Table 1 presents recommendations for the administration of these oral medications via enteral feeding tube. Additionally, nursing staff should be given detailed instructions regarding proper enteral drug administration procedures [7].

**Table 1:** **Recommendations for enteral administration of pulmonary arterial hypertension medications**

**Drug**	**Oral suspension available**	**Intravenous formulation available**	**Data supporting enteral drug administration**
Sildenafil [8–10]	Yes	Yes (dosage is 10 mg every 8 hours)	Reports exist where sildenafil tablet was crushed, dissolved in 5 ml sterile water and administered via enteral tube
Tadalafil [11,12]	No^a^	No	No data available. May be reasonable to use extemporaneous preparation of tadalafil suspension for enteral administration
Bosentan [13–16]	No^a^	No	Institute for Safe Medication Practices does *not* recommend crushing due to teratogenic properties^b^
Ambrisentan [14,17]	No	No	Institute for Safe Medication Practices: ‘do not crush medication’ (extended release product with teratogenic properties); do *not* administer via enteral tube
Macitentan [18]	No	No	Manufacturer recommends that tablets *not* be split, chewed or crushed; do *not* administer via enteral tube
Riociguat [19]	No	No	No data available; would *not* recommend crushing due to teratogenic properties

^a^May be compounded as follows: grind 15 tadalafil 20 mg tablets in a glass mortar, then mix with 30 ml Ora-Plus (Paddock Laboratories, Minneapolis, MN, USA) and 30 ml Ora-Sweet (Paddock Laboratories) to make a final volume of 60 ml. Suspension is stable for at least 91 days when stored in amber plastic bottles at room temperature. ^b^May be compounded as follows: place bosentan tablet in 5 to 25 ml water to create a suspension. An appropriate aliquot of the suspension can be used to deliver the prescribed dose. Any remaining suspension should be discarded. Bosentan should not be mixed or dissolved in liquids with a low (acidic) pH (for example, fruit juices) due to poor solubility; the drug is most soluble in solutions with pH >8.5. Women who are or may become pregnant should not handle crushed bosentan.

Administration of inhaled outpatient therapy is problematic when patients require mechanical ventilation. Products such as iloprost (Ventavis®; Actelion Pharmaceuticals, San Francisco, CA, USA) and treprostinil (Tyvaso®; United Therapeutics, Research Triangle Park, NC, USA) require delivery with specialized delivery devices: iloprost, I-neb® Adaptive Aerosol Delivery® (Philips Healthcare, Andover, MA, USA) or Prodose® Adaptive Aerosol Delivery® (Philips Healthcare) [20]; and treprostinil, Tyvaso inhalation system® (United Therapeutics) [21]. Although reports exist of successful inhaled iloprost delivery to patients requiring mechanical ventilation or high-flow oxygen [22], this delivery method has not been extensively tested, nor is it approved by the US Food and Drug Administration. Furthermore, patients requiring mechanical ventilation may benefit from a titratable medication that is delivered continuously rather than intermittently. Hence continuous administration of pulmonary vasodilator therapy (nebulized epoprostenol or inhaled nitric oxide) may be advantageous because of the ability to titrate the dose and the likelihood that continuous drug delivery will be less likely to alter hemodynamics compared with intermittent drug delivery (see Altered mental status section for more on inhaled nitric oxide and nebulized epoprostenol).

## Bacteremia and sepsis

The incidence of bacteremia in patients with PAH is elevated compared with that in the general public. This is secondary to the central venous catheters used to deliver intravenous prostanoid therapy [23]. The incidence of bacteremia has been estimated at 0.118 episodes per 1,000 treatment-days in patients receiving epoprostenol and as 0.938 episodes per 1,000 treatment-days in patients receiving treprostinil [24]. Although improvements in administration techniques may further decrease the incidence of bacterial infection associated with treprostinil therapy, bloodstream infections may still occur [25]. When a patient develops bacteremia with or without sepsis there are difficult questions that arise.

First, it is important to address whether or not a tunneled catheter used for prostacyclin infusion should be removed in the setting of bacteremia or sepsis. We agree with current Infectious Diseases Society of America guidelines that any patient with sepsis should have the indwelling catheter removed after alternate access is obtained and the prostacyclin infusion is transferred [26]. We also feel that patients with known bacteremia and tachycardia, even if not fulfilling the criteria for sepsis, should have consideration of catheter removal. This is due to the risk that tachycardia and systemic vasodilatation from bacteremia may pose to the right ventricle that is already strained from PAH. If a patient is bacteremic but normotensive and without tachycardia, then culture results can be awaited while antibiotics are administered, since infections with coagulase-negative staphylococcus and enterococcus may not necessitate catheter removal [26]. If a decision is made to remove the catheter, alternative access must be obtained prior to catheter removal to ensure continuous prostacyclin delivery. Central venous access (including peripherally inserted central catheters) is preferred over peripheral intravenous lines, due to the less stable nature of peripheral lines. If the inserted central catheter has multiple lumens, we recommend labeling or marking the lumen through which prostacyclin will infuse. In this way, the possibility of manipulating the prostacyclin line will be minimized. In general, once a tunneled catheter is removed due to infection with bacteremia, it should not be replaced until the patient has completed a full course of antibiotics and has negative blood cultures. Replacement of the tunneled catheter is generally done in the outpatient setting after recovery.

Second, if the central venous catheter associated with prostacyclin infusion is a potential source of bacteremia and needs to be removed, how should the prostacyclin infusion be managed? Possible solutions to this question include: rapidly transferring the infusion from the old catheter to the new catheter without priming the new catheter (discouraged in most cases, see below); priming the new catheter with medication prior to rapid transference of the infusion to the new catheter; or overlapping infusions, with one running through the old catheter until a volume is instilled in the new catheter that approximates the volume of that catheter. Variables involved in this decision include the dead space present in the catheter (Table 2), the pump infusion rate (Table 3), the half-life of the infusing medication and the patient’s condition [27–29].

**Table 2:** **Volumes associated with various catheter types**

**Catheter type**	**Catheter volume**
55 cm PICC line, 17 gauge lumen	0.76 ml [28]
55 cm PICC line, 18 gauge lumen	0.56 to 0.68 ml [28]
55 cm PICC line, 19 gauge lumen	0.44 ml [28]
Single-lumen Hickman catheter, 9.6 French	1.8 ml [28]
Double-lumen Hickman catheter, 9 French	0.6 ml (small port), 1.3 ml (large port) [28]
Triple-lumen catheter	0.38 ml (18 gauge ports), 0.42 ml (16 gauge ports) [29]

PICC, peripherally inserted central catheter.

**Table 3:** **Flow rates associated with intravenous infusion pumps**

	**CADD legacy pump** ^**a**^	**CADD-MS3 pump** ^**a**^	**CRONO five pump** ^**b**^	**Alaris IV pump** ^**c**^
Epoprostenol rate range (ml/hour)	0.01 to 3.6	Not applicable	Not Applicable	0.1 to 999 [27]
Treprostinil rate range (ml/hour)	0.01 to 3.6	0.002 to 1.00	0.05 to 5	0.1 to 999 [27]

^a^Smiths Medical, St Paul, MN, USA. ^b^Intra Pump Infusion Services, Grapevine, TX, USA. ^c^CareFusion, San Diego, CA, USA. CADD, computerized ambulatory drug delivery.

While Table 2 presents a guide for the volume of various catheters, Hickman catheters are often cut to patient length and may have a different volume than anticipated. Measuring the cut catheter volume with saline prior to insertion can provide the volume of the catheter in this instance. While several centers have a policy of priming 0.75 ml medication from a computerized ambulatory drug delivery (CADD) Legacy pump (Smiths Medical, St Paul, MN, USA) into the new catheter before rapid sequence transfer of medication (personal communication with other pulmonary hypertension practitioners and specialty pharmacies), this does not account well for the above variables. If a treprostinil infusion is transitioned from a Hickman catheter to a peripherally inserted central catheter line, a nontunneled central venous catheter, or a peripheral intravenous catheter, then a rapid transition without priming is usually well tolerated, as the medication will be delivered systemically prior to a half-life elimination. This is contrasted with a transition from a peripherally inserted central catheter line to a single lumen Hickman catheter. Because of the large catheter volume of the Hickman catheter, the time from initiation of infusion to systemic delivery could be as long as 90 hours (for a slowly infusing treprostinil infusion from a CADD-MS3 pump (Smiths Medical)), much longer than the 4.5-hour half-life of the medication. Similarly, a patient receiving intravenous therapy (epoprostenol or treprostinil) from a CADD Legacy pump could have a delay from initiation to systemic delivery of 1 to 2 hours, much longer than the half-life of 3 to 6 minutes. In both examples, consideration must be given to either overlapping the infusions or priming the new catheter with medication, so that excessive time without medication can be avoided. Of these two options, we prefer overlapping medication, because this will prevent the potentially ill-effects of inadvertent drug bolus administration while priming the new line (in our experience, even a bolus of one-half the catheter volume instilled over 5 to 10 minutes can lead to symptoms of prostacyclin toxicity). We believe that symptoms of prostacyclin excess can occur with catheter priming due to rapid instillation of product into the catheter causing unintended transference of medication systemically.

Third, should the patient remain on their existing infusion pump, or should the patient be transitioned to a hospital pump? There are potential advantages and pitfalls to each approach. If the patient remains on their pump, the hospital staff may be unfamiliar with the pump and unable to alter the infusion. This places the responsibility of managing the infusion pump on the patient or their mixing partner. If the patient is critically ill and in an unusual environment, they may be more prone to medication error. If the pump is under the care of a mixing partner, they may be unavailable to change the medication when needed, and user error is again a concern. However, if the decision is made to change the infusion to a hospital pump, a change in medication concentration may be required due to limitations in infusion rates on the hospital pump (see Table 3). A change in medication concentration could lead to wide changes in drug delivery to the patient. For example, if a patient weighing 60 kg usually receives 100 ng/kg/minute treprostinil (10 mg/ml) delivered by CADD-MS3 pump at a rate of 0.036 ml/hour through a Hickman catheter with volume of 1.8 ml, and they are changed to a hospital pump requiring a change in treprostinil concentration to 1 mg/ml treprostinil so that an equivalent dose can be maintained at a rate of 0.36 ml/hour, then the first 5 hours of infusion through the hospital pump will infuse the remaining 10 mg/ml treprostinil indwelling in the catheter, which could deliver a dose 10 times that intended.

Even among pulmonary hypertension centers, the decision of whether to transition from patient pump to hospital pump is debatable and clinical practice varies significantly [30]. In our institution, all medication is supplied from pharmacy with mixing instructions checked among members of our PAH team from nursing, pharmacy and physicians. On our pulmonary hypertension unit, where all nurses are regularly in-serviced on pumps and are familiar with infusions, patients may stay on their own pump with nursing assisting with management. In our ICU, where nurses may have little prostacyclin experience, patients on CADD Legacy pumps are transitioned to hospital pumps, as the rate and concentration of infusion can often be left unchanged. However, patients on CADD-MS3 pumps in our ICU are not transitioned, due to the inability to match the rate and concentration on hospital pumps.

As infused prostacyclin therapy is intricate in its delivery, and errors in management could have significant deleterious effects, we recommend that management of inpatients on prostacyclin therapy be managed at pulmonary hypertension centers. This ensures a multidisciplinary approach in which pharmacy, nursing and practitioners have significant experience to minimize error. It is also important to know that specialty pharmacies which deliver prostacyclin therapy have nursing experiences with pump therapy available at all times to help manage issues with pump therapy.

Fourth, how should oral or inhaled medication be managed in patients with hypotension related to sepsis? If a patient develops hypotension while on treatment for PAH, then clinical management must be individualized. The concern is that the pulmonary vasodilators used to treat PAH cause some systemic vasodilatation. In the setting of hypotension from sepsis, continued use of medications that potentiate hypotension could decrease perfusion and lead to further end-organ dysfunction. We would rarely recommend that a prostacyclin infusion be altered or decreased, even if vasopressor support is indicated. If prostacyclin infusion is decreased in an attempt to improve systemic blood pressure, this should be done under the direction of a practitioner with extensive prostacyclin experience. If a patient is on oral medication and becomes hypotensive, the decision to hold these medications depends on certain variables. The risk of acutely stopping these medications is difficult to determine. Factors such as the degree of hypoxemia, the extent of right ventricular dysfunction and whether the right ventricle is in compensated or decompensated failure may affect management. This must be balanced with the risk of continuing oral medications, because they can potentiate hypotension and cause a resultant decrease in organ perfusion in the setting of sepsis. In the absence of significant right ventricular failure in a patient with evidence of end-organ hypoperfusion, then consideration of dose decrease (if possible) or even cessation of oral medication is necessary.

## Altered mental status

Delirium and sedation are common in the ICU setting, affecting up to 82% of ICU patients [31]. Altered mental status can have a significant impact on medication delivery of patients with PAH. Oral medications can usually be managed as described in Table 1. Pump infusion can become more complicated, especially if the patient has been involved in the process of mixing and pump management. When a patient is unable to care for a pump on their own, and if they are far away from their home, it may be difficult to rely on their mixing partner to care for the infusion. Therefore, it is essential that the healthcare system develops a plan to care for the infusion, or quickly transfer the patient to a facility with more experience, as described in the Bacteremia and sepsis section above.

If a patient is on an inhaled therapy and develops altered mental status, they will be unable to use this medication as it requires patient participation for drug delivery. An alternative mode of delivery therefore needs to be established. At our institution, this alternative typically involves transition to continuous delivery of either inhaled epoprostenol or inhaled nitric oxide. While neither therapy has been approved by the US Food and Drug Administration for ICU use in adult patients within the United States, they both have established hemodynamic effects in patients with PAH, and one has not been shown superior to the other [32]. Because of concerns associated with possible side effects from inhaled nitric oxide (nitrogen dioxide accumulation and methemoglobinemia [33]), as well as the high cost of therapy, our institution usually initiates inhaled epoprostenol.

**Figure 1:**
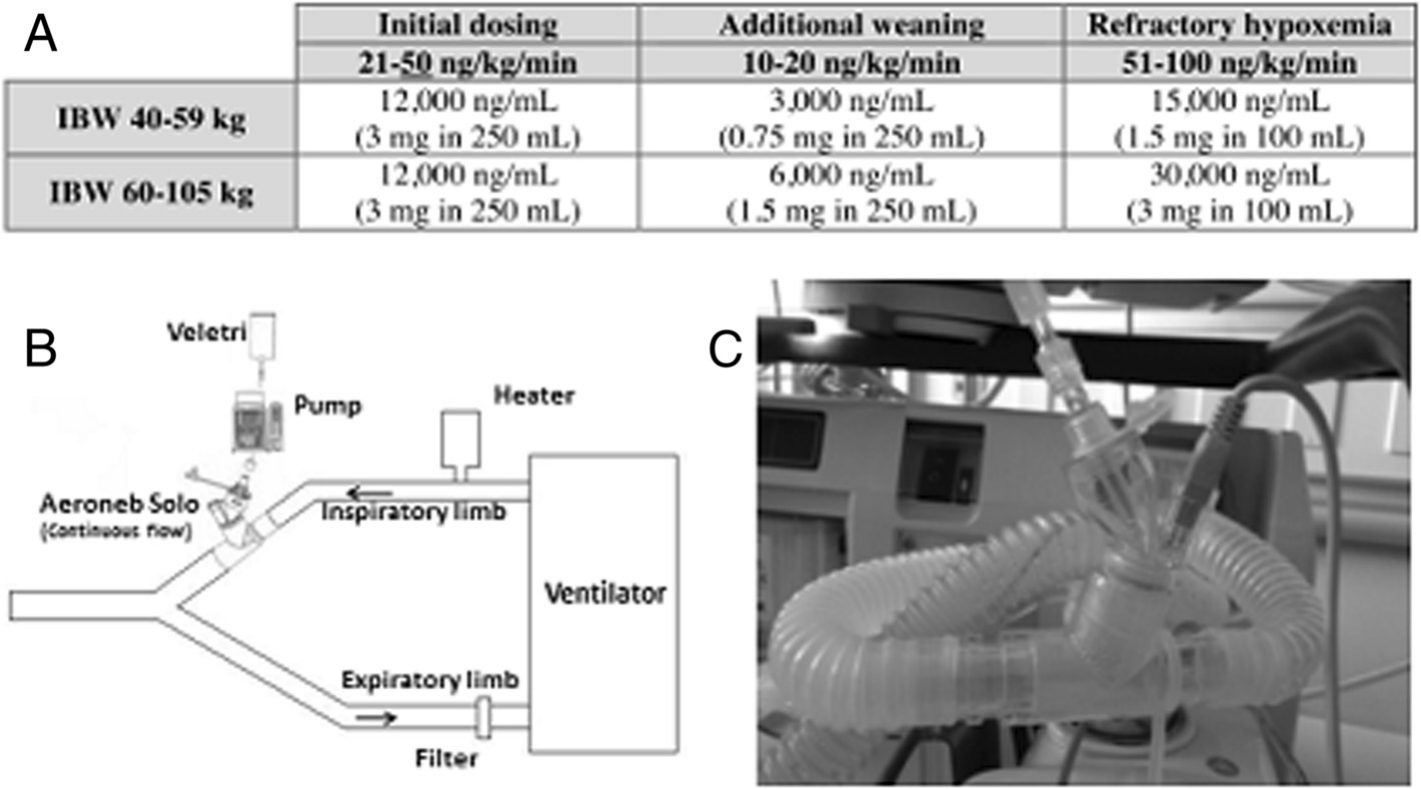
**Standardization of inhaled epoprostenol in our electronic record system and the Aeroneb method of delivery. (A)** Dosage of inhaled epoprostenol as ordered through our electronic medical record. Concentrations and dosages selected to minimize bag changes and to improve safety. IBW, ideal body weight. **(B)** Left: our set-up for delivering inhaled epoprostenol through an aeroneb on patients with mechanical ventilation. Right: the Aeroneb (Aerogen Ltd, Dangan, Galway, Ireland) in relation to tubing placed after the ventilator filter. This position was found with repeated trial and error to optimize aerosol delivery without significant deposition in ventilator tubing.

The optimal dose of inhaled epoprostenol for adult patients with pulmonary hypertension is unknown; however, an inverse dose–response relationship exists between the inhaled epoprostenol dose and pulmonary artery pressures [34]. Dosing of inhaled epoprostenol reported in the primary literature is variable and ranges from 5 to 85 ng/kg/minute [35]. At our institution, we generally start at a dose of either 50 or 100 ng/kg/minute and titrate by oxygen saturation values and pulmonary artery pressures (measured by echocardiogram), where our maximum dose is 100 ng/kg/minute and our minimum dose is 10 ng/kg/minute. As epoprostenol has an extremely short half-life, dose adjustments are made as quickly as every 15 minutes. The duration of therapy is variable and is based on patient improvement and the ability to resume or up-titrate other therapies. While most patients only require nebulized epoprostenol as a temporary bridge, durations of therapy exceeding 2 weeks have been reported [35]. We have also worked to eliminate safety events by standardizing ordering of inhaled epoprostenol in our electronic record system (Figure 1A) and by selecting the Aeroneb (Aerogen Ltd, Dangan, Galway, Ireland) method of delivery [36] to prevent any loss of airflow from nebulized delivery of medication (Figure 1B). At our institution, we have used either inhaled nitric oxide or inhaled epoprostenol in the operating room and ICU settings for patients who are unable to receive inhaled prostacyclin therapies.

## Need for gastrointestinal rest

There are many conditions in the ICU during which a patient may not be able to take medications enterally (*nil per os*). These include but are not limited to perioperative care, acute cholecystitis, acute pancreatitis and bowel obstruction. When a patient is *nil per os*, they can typically continue pump therapy or inhaled therapy without change if their mental status is intact. The challenge is whether or not oral medications require a transition to other forms of therapy. Sildenafil is available intravenously, and may be administered instead of oral dosing if given at 50% of the typical oral dose [8]. Other oral medications are not available intravenously, and consideration of alternate therapies is needed. The current symptoms of the patient, as well as the estimated length a patient may be *nil per os*, may influence whether a patient requires alternate PAH treatment for an interval. If patients require a transition, we often begin with inhaled delivery of inhaled nitric oxide or inhaled epoprostenol (see Altered mental status section above for details). As with all PAH patients, evidence of right ventricular decompensation may necessitate a transition to pump therapy.

## Acute renal insufficiency/failure

Renal failure is prevalent among patients admitted to the ICU. A study from September 2000 to December 2001 identified a point prevalence rate for developing acute renal failure in the ICU ranging from 1.4 to 25.9%, where the average point prevalence rate was 5.7% [37]. The risk may be higher in PAH, as one study determined that 34% of PAH patients hospitalized for right heart failure developed worsening renal function during hospitalization (defined as a rise in creatinine of 0.3 mg/dl within the first 48 hours of admission) [38]. This phenomenon is probably multifactorial and potential etiologies include venous congestion, hypotension (either from decreased cardiac output or adverse medication effects) and the use of nephrotoxic agents (for example, intravenous contrast, furosemide, and so forth). Patients with PAH and acute renal failure may be at risk for clinical deterioration from fluid overload and resultant right heart strain. The provider must balance the patient’s pulmonary status with the hemodynamic side effects of PAH pharmacotherapy. Data regarding use of phosphodiesterase type 5 inhibitors, endothelin-receptor antagonists, intravenous prostacyclin therapy, soluble guanylate cyclase stimulator and inhaled vasodilator therapy in renal insufficiency and failure are summarized in Table 4.

**Table 4:** **Monitoring and dosage of pulmonary arterial hypertension pharmacotherapy in renal insufficiency**

**Drug**	**Degree of renal elimination**	**Dosage adjustments**	**Monitoring**	**Recommendations**
**Phosphodiesterase type 5 inhibitors**				
Sildenafil [8]	Nonrenal metabolism to active metabolites; 13% of dose excreted in urine as metabolites	No dose adjustment is required. In patients with CrCl ≤30 ml/minute, exposure to parent drug and active metabolite increased twofold	Worsening of side effects (hypotension, epistaxis, nasal congestion, headache, dyspepsia, flushing, insomnia, erythema, dyspnea and/or rhinitis)	Monitor for worsening of side effects and decrease dosage if indicated.
Tadalafil [11]	Nonrenal metabolism; however, renal impairment results in twofold to fourfold increase in tadalafil exposure	CrCl 31 to 80 ml/minute: start dosing at 20 mg once daily; increase to 40 mg once daily based on individual tolerability. CrCl ≤30 ml/minute or hemodialysis: avoid use.	Worsening of side effects (hypotension, headache)	Monitor for hypotension, consider dose reduction. Severe impairment: hold dose if side effects worsened
**Endothelin-receptor antagonists**				
Ambrisentan [17]	Nonrenal elimination	No adjustment for mild–moderate renal failure; severe renal failure and hemodialysis not studied	Fluid overload	Hold if fluid overload is problematic
Bosentan [13]	<3% eliminated in urine	No adjustment necessary	Fluid overload	Hold if fluid overload is problematic
Macitentan [13]	Nonrenal elimination	No adjustment necessary	Worsening of side effects (anemia, nasopharyngitis, pharyngitis, bronchitis, headache, influenza, and urinary tract infection)	Likely safe to use in patients with renal dysfunction
**Soluble guanylate cyclase stimulator**				
Riociguat [19]	40% drug eliminated in urine (mostly as inactive metabolites)	Not recommended in patients with CrCl <15 ml/minute or those receiving dialysis	Possible hypotension bleeding or other side effects (headache, dizziness, dyspepsia/gastritis, nausea, diarrhea, hypotension, vomiting, anemia, gastroesophageal reflux, and constipation)	Monitor for hypotension, consider dose reduction. Severe impairment (anuria): hold dose if side effects worsened
**Intravenous prostacyclin therapy**				
Epoprostenol [39,40]	Nonrenal elimination; metabolites recovered in urine, some of which are minimally active	No adjustment necessary; if initiating therapy, consider starting at a low dose and titrating slowly	Possible hypotension or side effects (headache, jaw pain, flushing)	Titrate slowly during initiation phase; consider gradual dosage reduction if patient is hypotensive and experiencing increased side effects (flushing, headache, jaw pain); do not abruptly discontinue therapy
Treprostinil [41]	4% of unchanged drug eliminated in urine; all metabolites (inactive) renally eliminated	No specific recommendation available	Possible hypotension or side effects (headache, jaw pain, flushing)	Titrate slowly during initiation phase; consider gradual dosage reduction if patient is hypotensive and is experiencing increased side effects (flushing, headache, jaw pain); do not abruptly discontinue therapy
**Inhaled vasodilators (prostacyclin and nonprostacyclin)**				
Inhaled nitric oxide [42]	Combines with oxyhemoglobin to produce methemoglobin and nitrate; nitrate is renally eliminated	No dosage adjustments recommended/required	Methemoglobin	Probably safe to use in patients with renal dysfunction
Treprostinil, inhaled [21]	4% of unchanged drug eliminated in urine; all metabolites (inactive) renally eliminated	No specific recommendation available	Possible hypotension or side effects (headache, jaw pain, flushing)	Titrate slowly during initiation phase; consider increasing dosing interval if patient is hypotensive and experiencing increased side effects (flushing, headache, jaw pain)
Iloprost, inhaled [20]	Nonrenal elimination	Not studied in patients with renal impairment; based on drug elimination, accumulation not expected	Possible syncope or side effects (headache, flushing, dizziness, nausea, vomiting or diarrhea)	Probably safe for use in patients with renal dysfunction. Continue usual monitoring

CrCl, creatinine clearance.

## Acute hepatic impairment/failure

Liver injury is common among critically ill patients. At ICU admission, liver function tests are found to be abnormal in as many as 61% of patients [43]. Cholestasis (as determined by a bilirubin level >2 mg/dl) occurs in 20% of ICU patients during their ICU admission [44]. Additionally, hepatocellular injury in the ICU occurs frequently, with hypoxic liver injury identified in up to 10% of patients admitted to a medical ICU [45]. Other causes of hepatocellular injury in critically ill patients include congestive hepatopathy, septic shock and drug-induced liver damage [46]. Patients with PAH are prone to developing congestive hepatopathy due to the likelihood of right ventricular failure in this population. Furthermore, medications such as bosentan have been implicated in drug-induced hepatic injury, including cases of severe hepatotoxicity [13,47]. The clinical impact of liver injury on patients with PAH can be significant, as most medications used to treat PAH undergo hepatic metabolism. Liver injury can impair drug metabolism and result in drug toxicity. Furthermore, medications such as bosentan have been implicated in drug-induced hepatic injury, including cases of severe hepatotoxicity [13,47]. Table 5 describes the hepatic metabolism of phosphodiesterase type 5 inhibitors, endothelin-receptor antagonists, soluble guanylate cyclase stimulator, intravenous prostacyclin therapy and inhaled vasodilator therapy, and provides recommendations for the clinician regarding dosage adjustment and monitoring parameters.

**Table 5:** **Monitoring and dosage of pulmonary arterial hypertension pharmacotherapy in hepatic impairment**

**Drug**	**Degree of hepatic elimination**	**Dosage adjustments**	**Monitoring**	**Recommendations**
**Phosphodiesterase type 5 inhibitors**				
Sildenafil [8]	Extensive hepatic metabolism (CYP3A4 major; CYP2C9 minor); active metabolite (which is 50% ==as active as sildenafil) also undergoes hepatic metabolism	No dose adjustment for mild to moderate impairment is required. Severe impairment has not been studied	Hypotension; worsening of side effects (epistaxis, headache, dyspepsia, flushing, insomnia, erythema, dyspnea and/or rhinitis)	Hold or reduce dose if patient presents with acute liver injury and hypotension.
Tadalafil [11]	Hepatic metabolism via CYP3A to inactive metabolites (which undergo methylation and glucuronidation)	Child–Pugh class A or B: reduce starting dose to 20 mg once per day	Hypotension; worsening of side effects (headache)	Hold or reduce dose if patient presents with acute liver injury and hypotension
		Child–Pugh class C: avoid use		
**Endothelin-receptor antagonists**				
Ambrisentan [17]	Hepatic metabolism via CYP3A, CYP2C19, and UGTs 1A9S, 2B7S an 1A3S; substrate OATP1B1 and OATP1B3; substrate but not an inhibitor of P-glycoprotein	Not recommended in patients with moderate or severe hepatic impairment	Peripheral edema/fluid overload, nasal congestion, sinusitis and/or flushing	Package labeling recommendation: discontinue if aminotransferase elevations >5× ULN or if elevations are accompanied by bilirubin >2× ULN, or by signs or symptoms of liver dysfunction and other causes are excluded
Bosentan [13]	Eliminated by biliary excretion following metabolism in the liver; three metabolites (one active); induces CYP2C9, CYP34 and possibly CYP2C19. Thought to induce its own metabolism	Child–Pugh class A: no dosage adjustment required	Peripheral edema/fluid overload, anemia, respiratory tract infections	Package labeling: ALT/AST >5× and <8× ULN, stop treatment and monitor ALT/AST levels at least every 2 weeks. Once the ALT/AST levels return to pretreatment values, consider reintroduction of the treatment
		Child–Pugh class B or C: avoid use		ALT/AST >8× ULN: discontinue treatment indefinitely
Macitentan [13]	Metabolized primarily by oxidative depropylation of the sulfamide to form the pharmacologically active metabolite. This reaction is dependent on CYP3A4 (major) and CYP2C19 (minor)	No adjustment necessary	Anemia, nasopharyngitis, pharyngitis, bronchitis, headache, influenza, and urinary tract infection	Package labeling: if clinically relevant aminotransferase elevations occur, or if elevations are accompanied by an increase in bilirubin >2× ULN, or by clinical symptoms of hepatotoxicity, discontinue macitentan. Consider reinitiation when hepatic enzyme levels normalize in patients who have not experienced clinical symptoms of hepatotoxicity
**Soluble guanylate cyclase stimulator**				
Riociguat [19]	Metabolized by CYP1A1, CYP3A, CYP2C8 and CYP2J2. Formation of the major active metabolite, M1, is catalyzed by CYP1A1; M1 is further metabolized to the inactive N-glucuronide.	Child–Pugh class A or B: no dosage adjustment required. Child–Pugh class B or C: no data available	Hypotension, bleeding or other side effects (headache, dizziness, dyspepsia/gastritis, nausea, diarrhea, hypotension, vomiting, anemia, gastroesophageal reflux, and constipation)	Hold or reduce dose if patient presents with significant acute liver injury and hypotension
**Intravenous prostacyclin therapy**				
Epoprostenol [39,40]	Rapid metabolism via hydrolysis at neutral pH in blood (major), also subject to enzymatic degradation (minor); two minimally active metabolites (one from hydrolysis, one from enzymatic degradation)	No adjustment necessary; if initiating therapy, consider starting at the low end of the dosing range and titrating slowly	Possible hypotension or side effects (headache, jaw pain, flushing)	Probably safe for use in patients with liver dysfunction; consider dosage reduction if patient is hypotensive and is experiencing increased side effects (flushing, headache, jaw pain); do not abruptly discontinue therapy
Treprostinil [41]	Hepatic metabolism via CYP2C8 (major) and via oxidation and glucuronidation (minor)	Mild to moderate hepatic insufficiency: initial dose should be decreased to 0.625 ng/kg/minute ideal body weight; cautious dosage increase	Hypotension or side effects (headache, jaw pain, flushing)	Exposure is increased in patients with hepatic insufficiency; if patient presents with acute liver injury and signs of increased drug exposure (hypotension, headache, flushing, jaw pain, and so forth), cautiously and gradually decrease dosage until symptoms subside. Do not abruptly discontinue therapy. One reasonable approach is to decrease the treprostinil dose by 10% every 3 hours until symptoms improve
		Severe hepatic insufficiency: no studies performed		
**Inhaled vasodilators**				
Nitric oxide, inhaled [42]	Nonhepatic elimination	No dosage adjustments recommended/required	Methemoglobin	Probably safe to use in patients with hepatic dysfunction
Treprostinil, inhaled [21]	Hepatic metabolism via CYP2C8 (major) and via oxidation and glucuronidation (minor)	Mild–moderate hepatic impairment: up-titrate slowly when initiating. Severe hepatic insufficiency: no studies performed	Hypotension or worsening of side effects (headache, jaw pain, flushing)	Exposure is increased in patients with hepatic insufficiency; if patient presents with acute liver injury and signs of increased drug exposure (hypotension, headache, flushing, jaw pain, and so forth), increase dosing interval and/or decrease inhalations per treatment until symptoms subside
Iloprost, inhaled [20]	Hepatic metabolism via β-oxidation (major) and cytochrome P450 (minor); major metabolite is inactive	Child–Pugh class B or C: consider increasing the dosing interval (for example, 3 to 4 hours between doses depending on the patient’s response at the end of the dose interval)	Hypotension/syncope, headache, flushing, ALP/GGT increased, flu-like symptoms, hemoptysis, muscle pain/cramping	Exposure is increased in patients with hepatic insufficiency; if patient presents with acute liver injury and signs of increased drug exposure (hypotension, headache, jaw pain, and so forth), consider increasing the dosing interval until symptoms subside

ALP, alkaline phosphatase; ALT, alanine aminotransferase; AST, aspartate aminotransferase; GGT, gamma-glutamyl transpeptidase; OATP, organic anion transporting polypeptide; UGT, uridine 5′-diphosphate glucuronosyltransferase; ULN, upper limit of normal.

## Conclusions

The care of patients with PAH during critical illness is complex due to their underlying disease and the nature of pulmonary vasodilator therapy. This complexity is most apparent in patients on prostacyclin therapy. As patients with PAH live longer, the chance of admission for critical illness will increase. During critical illness, managing PAH medications can be very difficult, and consequences of mistakes can be severe. After years of managing patients with PAH through their critical illness, we have learned some practices that are important to avoid and some practices that have generally helped us to navigate through difficult situations. There is little evidence to support our recommendations on this topic – this article is therefore not meant to be a practice guideline, because there is not sufficient evidence to support our practice. We would encourage further discussion, and standardization of care is encouraged for PAH patients on pulmonary vasodilators during emergencies.

## Abbreviations

CADD: Computerized ambulatory drug delivery
PAH: Pulmonary arterial hypertension
